# Correction: Cultivation and genomics of the first freshwater SAR11 (LD12) isolate

**DOI:** 10.1038/s41396-019-0569-7

**Published:** 2019-12-13

**Authors:** Michael W. Henson, V. Celeste Lanclos, Brant C. Faircloth, J. Cameron Thrash

**Affiliations:** 1 Department of Biological Sciences, Louisiana State University, Baton Rouge, LA 70803 USA; 2 Museum of Natural History, Louisiana State University, Baton Rouge, LA 70803 USA

**Keywords:** Water microbiology, Limnology, Microbial ecology, Bacterial genetics

**Correction to: The ISME Journal**

10.1038/s41396-018-0092-2

Since publication of the original article the authors noticed, regrettably, that the production of the article introduced errors into the nomenclature format for the organism, which were identified by the authors during proofing, but nevertheless remained uncorrected prior to publication of the article.

In the Abstract section “For strain LSUCC0530, we propose the provisional nomenclature Candidatus fonsibacter ubiquis” should read “For strain LSUCC0530, we propose the provisional nomenclature *Candidatus* Fonsibacter ubiquis”.

In the Discussion section For the first cultured representative of the LD12 clade, we propose the provisional taxonomic assignment for strain LSUCC0530 as “*Candidatus fonsibacter ubiquis*” should read For the first cultured representative of the LD12 clade, we propose the provisional taxonomic assignment for strain LSUCC0530 as “*Candidatus* Fonsibacter ubiquis”.

Furthermore, the authors realised they mistakenly used “dehydrogenase” instead of “lyase” in the abstract: “The genome affirms many previous metabolic predictions from cultivation-independent analyses, like a complete Embden–Meyerhof–Parnas glycolysis pathway, but also provides novel insights, such as the first isocitrate dehydrogenase in LD12” should read “The genome affirms many previous metabolic predictions from cultivation-independent analyses, like a complete Embden–Meyerhof–Parnas glycolysis pathway, but also provides novel insights, such as the first isocitrate lyase in LD12”.

The publisher and authors apologise for any inconvenience caused.

