# Supplementary material for: Cultivation and genomics of the first freshwater SAR11 (LD12) isolate
Source: ISME J. 2018 Mar 29;12(7):1846–60. doi: 10.1038/s41396-018-0092-2 (PMC6018831; doi:10.1038/s41396-018-0092-2)
Supplement: Supplementary file 1 — Supplemental Text(DOCX 358 kb) [file 41396_2018_92_MOESM1_ESM.docx]

**Supplemental Information** for:

Cultivation and genomics of the first freshwater SAR11 (LD12) isolate

Michael W. Henson^1^, V. Celeste Lanclos^1^, Brant C. Faircloth^1,2^, and J. Cameron Thrash^1,3^

1. Department of Biological Sciences, Louisiana State University, Baton Rouge, LA 70803, U.S.A.

2. Museum of Natural History, Louisiana State University, Baton Rouge, LA 70803, U.S.A.

3. Materials and Correspondence:

J. Cameron Thrash

Department of Biological Sciences

202 Life Sciences Bldg.

Louisiana State University

Baton Rouge, LA 70803

thrashc@lsu.edu

225-578-8210

**Supplemental Text**

***Genome assembly and quality assessment***

**I. SPAdes assembly**

**Step 1.** Subset the reads.

$ seqtk sample -s100 530_R1.fastq 1000000 > 530_subset1.r1.fastq

$ seqtk sample -s100 530_R2.fastq 1000000 > 530_subset1.r2.fastq

Adapter file for Trimmomatic (Bolger *et al.*, 2014) :

>i7

GATCGGAAGAGCACACGTCTGAACTCCAGTCACAGGTTCGAATCTCGTATGCCGTCTTCTGCTTG

>i5

AGATCGGAAGAGCGTCGTGTAGGGAAAGAGTGTCTCCTAGAGTGTAGATCTCGGTGGTCGCCGTATCATT

**Step 2.** Trim pretty aggressively to remove adapters and bases < q20 at ends and over sliding window

$ java -jar ~/bin/trimmomatic-0.30.jar PE -threads 12 -phred33 -trimlog \

trimmed/530_trimlog.log \

untrimmed/530_subset1.r1.fastq \

untrimmed/530_subset1.r2.fastq \

trimmed/530_subset1.r1.fastq \

trimmed/530_subset1.r1.unpaired.fastq \

trimmed/530_subset1.r2.fastq \

trimmed/530_subset1.r2.unpaired.fastq \

ILLUMINACLIP:/scratch/jcthrash-test/530_adapters.fa:2:30:10 \

LEADING:20 TRAILING:20 SLIDINGWINDOW:13:20 MINLEN:40

**Step 3.** Assemble w/ SPAdes (Bankevich *et al.*, 2012):

$ python ~/src/SPAdes-3.10.1-Linux/bin/spades.py --threads 12 --memory 46 --cov-cutoff auto \

--pe1-1 trimmed/530_subset1.r1.fastq \

--pe1-2 trimmed/530_subset1.r2.fastq \

--s1 trimmed/530_subset1.r1.unpaired.fastq \

--s2 trimmed/530_subset1.r2.unpaired.fastq \

-o 530_assembly

This resulted in a single contig with overlapping ends, plus three very small sequences. The first was 83 bp and contained only repeats of “CCCTAA, ” the second was 78 bp of “C,” the third, 78 bp of “A.”

**II. Quality assessment**

The single scaffold from the original SPAdes assembly was evaluated with Reapr (Hunt *et al.*, 2013), manually broken based on the results (two FCD errors), and the gaps were extended using SSPACE (Boetzer *et al.*, 2011) and all the Illumina HiSeq reads (Step 1). Overlaps were determined with megablast and the scaffolds were manually rejoined with overlaps removed. Another run of Reapr (Step 2) identified only one FCD error. The break and SSPACE extension process was repeated, and overlaps determined again with megablast. To evaluate the quality of the overlaps at the outer ends of the scaffold, we artificially broke the scaffold in two, reorganized the ends so they were now internal, and removed overlaps. Reapr did not identify any additional errors at this step, but verified the previous single FCD error (Step 3). The break-extension process was repeated one more time (Step 4) and then again using the MiSeq reads, with a final re-evaluation with reapr identified only the single FCD error in the same location (Step 5). To assess the validity of the error, we repeated the quality assessment of the final scaffold using a separate algorithm, Pilon (Walker *et al.*, 2014) after mapping all the HiSeq reads to the scaffold using BWA (Li and Durbin, 2009) (Step 6). This resulted in no errors. Corroborating information was obtained using CheckM (Parks *et al.*, 2015) (Step 7) and by evaluating the GC skew (Brown *et al.*, 2015) (Step 8). Detailed commands and outputs are as follows, according to the steps identified in this synopsis.

**Step 1.**

Reapr

$ reapr facheck 530_spades_sc.fasta

no error

$ reapr perfectmap 530_spades_sc.fasta 530_subset1.r1.fastq 530_subset1.r2.fastq 300 perfect

$ reapr smaltmap 530_spades_sc.fasta 530_subset1.r1.fastq 530_subset1.r2.fastq 530_bwa.bam

$ reapr pipeline 530_spades_sc.fasta 530_bwa.bam 530_reapr_output perfect

This gave similar output, with only two FCD errors and two small sections removed (and replaced with Ns). The scaffold was manually broken in these locations, with new fasta headers added, resulting in three scaffolds 🡪 04.break.broken_assembly_broken.fa.

SSPACE

$ perl SSPACE_Standard_v3.0.m.pl -l library.txt -s 04.break.broken_assembly_broken.fa -x 1 -v 1 -T 16 -b 530_sspacev2.1

The library.txt file specified all the original HiSeq reads. All three scaffolds were extended. Overlaps were identified with megablast by comparing each pair of scaffolds.

$ cat 530_sspacev2.1.final.scaffolds.fasta | fastaToTab | grep scaffold1 | tabToFasta > scaffold1.fasta

$ cat 530_sspacev2.1.final.scaffolds.fasta | fastaToTab | grep scaffold2 | tabToFasta > scaffold2.fasta

$ cat 530_sspacev2.1.final.scaffolds.fasta | fastaToTab | grep scaffold3 | tabToFasta > scaffold3.fasta

$ makeblastdb -dbtype nucl -in scaffold1.fasta -out scaffold1db -parse_seqids -hash_index

$ makeblastdb -dbtype nucl -in scaffold2.fasta -out scaffold2db -parse_seqids -hash_index

$ blastn -query scaffold2.fasta -db scaffold1db -out scaffold2v1blast

$ blastn -query scaffold3.fasta -db scaffold1db -out scaffold3v1blast

$ blastn -query scaffold3.fasta -db scaffold2db -out scaffold3v2blast

The order starting with the beginning of the longest scaffold is 1—>3—>2, with a loop back to 1. I removed overlaps and named the file 530_sspace_scaffold1.fasta.original.

To smooth out missing lines:

$ cat 530_sspace_scaffold1.fasta.original | fastaToTab | tabToFasta > 530_sspace_scaffold1.fasta

**Step 2.**

$ reapr facheck 530_sspace_scaffold1.fasta

no error

$ reapr perfectmap 530_sspace_scaffold1.fasta 530_R1.fastq 530_R2.fastq 300 perfect

$ reapr smaltmap -n 16 530_sspace_scaffold1.fasta 530_R1.fastq 530_R2.fastq 530_sspace_scaffold1.bam

$ reapr pipeline 530_sspace_scaffold1.fasta 530_sspace_scaffold1.bam 530_sspace_scaffold1_output perfect

Just one RCD error.

**Step 3.**

Check to see if that is the same and only error if the 2—>1 gap is closed. I’ve redone the scaffold assembly such that the scaffolds are 3—>2 —>1: 530_sspace_scaffold1_ro.fasta.original

$ cat 530_sspace_scaffold1_ro.fasta.original | fastaToTab | tabToFasta > 530_sspace_scaffold1_ro.fasta

$ reapr facheck 530_sspace_scaffold1_ro.fasta

no error

$ reapr perfectmap 530_sspace_scaffold1_ro.fasta 530_R1.fastq 530_R2.fastq 300 perfect

$ reapr smaltmap -n 16 530_sspace_scaffold1_ro.fasta 530_R1.fastq 530_R2.fastq 530_sspace_scaffold1_ro.bam

$ reapr pipeline 530_sspace_scaffold1_ro.fasta 530_sspace_scaffold1_ro.bam 530_sspace_scaffold1_ro_output perfect

The 04.break.broken_assembly_bin.fa files for both 530_sspace_scaffold1 and 530_sspace_scaffold1_ro are 100% identical across their alignments (NCBI megablast), but slightly different sizes. Importantly this also means that the scaffold end join is not an issue.

**Step 4.**

Now try to eliminate the FCD error with a final break and extension of 530_sspace_scaffold1.fasta.

Take the 04.break.broken_assembly.fa file from reapr_sspacev2.1a, manually remove the Ns, break the scaffold and rename the headers —> 04.break.broken_assembly_broken.fa.original

$ cat 04.break.broken_assembly_broken.fa.original | fastaToTab | tabToFasta > 530_sspace_scaffold1_broken.fasta

run SSPACE with the same library.txt file as above.

$ mv sspace.o630499 library.txt q_sspace 530_sspace_scaffold1_broken.fasta 530_sspacev2.2/

$ cd 530_sspacev2.2/

Although the scaffolds were not joined, the larger one was extended.

$ cat 530_sspacev2.2.final.scaffolds.fasta | fastaToTab | grep scaffold1 | tabToFasta > scaffold1.fasta

$ cat 530_sspacev2.2.final.scaffolds.fasta | fastaToTab | grep scaffold2 | tabToFasta > scaffold2.fasta

$ makeblastdb -dbtype nucl -in scaffold1.fasta -out scaffold1db -parse_seqids -hash_index

$ blastn -query scaffold2.fasta -db scaffold1db -out scaffold2v1blast

megablast showed that the beginning of scaffold two has a 616 bp overlap with the end of scaffold 1. Also, the end of scaffold 2 has a 462 bp overlap with the beginning of scaffold 1, as it should.

Join the two and run reaper another time —> 530_sspace_scaffold2.fasta.original

$ cat 530_sspace_scaffold2.fasta.original | fastaToTab | tabToFasta > 530_sspace_scaffold2.fasta

$ cp 530_sspace_scaffold2.fasta ../

$ cd ../

$ reapr facheck 530_sspace_scaffold2.fasta

no error

$ reapr perfectmap 530_sspace_scaffold2.fasta 530_R1.fastq 530_R2.fastq 300 perfect

$ reapr smaltmap -n 16 530_sspace_scaffold2.fasta 530_R1.fastq 530_R2.fastq 530_sspace_scaffold2.bam

$ reapr pipeline 530_sspace_scaffold2.fasta 530_sspace_scaffold2.bam 530_sspace_scaffold2_output perfect

This is still showing an FCD error in the same place. Attempt to close with MiSeq reads.

**Step 5.**

Manually break 04.break.broken_assembly.fa —> 04.break.broken_assembly_broken.fa.original.

$ cat 04.break.broken_assembly_broken.fa.original | fastaToTab | tabToFasta > 04.break.broken_assembly_broken.fa

Run SSPACE as above but with MiSeq reads.

This only extended one scaffold. Run megablast to identify overlaps:

$ cat 530_sspacev3.final.scaffolds.fasta | fastaToTab | grep scaffold1 | tabToFasta > scaffold1.fasta

$ cat 530_sspacev3.final.scaffolds.fasta | fastaToTab | grep scaffold2 | tabToFasta > scaffold2.fasta

$ makeblastdb -dbtype nucl -in scaffold1.fasta -out scaffold1db -parse_seqids -hash_index

$ blastn -query scaffold2.fasta -db scaffold1db -out scaffold2v1blast

There was the typical overlap from the scaffold ends, 462 bp in this case. There was only a 236 bp overlap between the end of scaffold 1 and the beginning of scaffold 2. I joined these manually for another reapr check —> 530_sspacev3_scaffold2.fasta.original

$ cat 530_sspacev3_scaffold2.fasta.original | fastaToTab | tabToFasta > 530_sspacev3_scaffold2.fasta

Run reapr with the miseq sequences and the hiseq sequences and compare FCD reports.

$ reapr facheck 530_sspacev3_scaffold2.fasta

no error

$ reapr perfectmap 530_sspacev3_scaffold2.fasta /project/jcthrash/genome_fastqs/LSUCC0530_S1_L001_R1_001.fastq /project/jcthrash/genome_fastqs/LSUCC0530_S1_L001_R2_001.fastq 440 perfect

$ reapr smaltmap -n 16 530_sspacev3_scaffold2.fasta /project/jcthrash/genome_fastqs/LSUCC0530_S1_L001_R1_001.fastq /project/jcthrash/genome_fastqs/LSUCC0530_S1_L001_R2_001.fastq 530_sspacev3_scaffold2.bam

$ reapr pipeline 530_sspacev3_scaffold2.fasta 530_sspacev3_scaffold2.bam 530_sspacev3_scaffold2_output perfect

This actually yielded no FCD errors, but many errors regarding low fragment coverage within the contig. Try running the hiseq reads across this new assembly.

$ reapr perfectmap 530_sspacev3_scaffold2.fasta /project/jcthrash/genome_fastqs/530_R1.fastq /project/jcthrash/genome_fastqs/530_R2.fastq 300 perfect

$ reapr smaltmap -n 16 530_sspacev3_scaffold2.fasta /project/jcthrash/genome_fastqs/530_R1.fastq /project/jcthrash/genome_fastqs/530_R2.fastq 530_sspacev3_scaffold2_hs.bam

$ reapr pipeline 530_sspacev3_scaffold2.fasta 530_sspacev3_scaffold2_hs.bam 530_sspacev3_scaffold2_hs_output perfect

This yielded the same erroneous region it did for all the checks (using all the reads). Time to cross-evaluate with a different algorithm.

**Step 6.**

Manually remove the scaffold overlaps at the ends of 530_sspacev3_scaffold2.fasta and change the header —> LSUCC0530_final_assembly.fasta

bwa

$ cd hiseq_bwa/

$ bwa index LSUCC0530_final_assembly.fasta

$ bwa aln -n 0 -t 16 LSUCC0530_final_assembly.fasta /project/jcthrash/genome_fastqs/530_R1.fastq > 530_R1.sai

$ bwa aln -n 0 -t 16 LSUCC0530_final_assembly.fasta /project/jcthrash/genome_fastqs/530_R2.fastq > 530_R2.sai

$ bwa sampe LSUCC0530_final_assembly.fasta 530_R1.sai 530_R2.sai /project/jcthrash/genome_fastqs/530_R1.fastq /project/jcthrash/genome_fastqs/530_R2.fastq > 530_R1R2.sam

$ samtools faidx LSUCC0530_final_assembly.fasta

$ samtools import LSUCC0530_final_assembly.fasta.fai 530_R1R2.sam 530_R1R2.bam

$ samtools sort 530_R1R2.bam 530_R1R2.sorted

$ samtools index 530_R1R2.sorted.bam

On my local machine

pilon

$ java -Xmx16G -jar ../../../Applications/pilon-1.22.jar --genome LSUCC0530_final_assembly.fasta --frags 530_R1R2.sorted.bam

Pilon version 1.22 Wed Mar 15 16:38:30 2017 -0400

Genome: LSUCC0530_final_assembly.fasta

Fixing snps, indels, gaps, local

Input genome size: 1160202

Scanning BAMs

530_R1R2.sorted.bam: 14264790 reads, 0 filtered, 9778615 mapped, 9537494 proper, 30284 stray, FR 100% 311+/-109, max 639

Processing LSUCC0530_final_assembly:1-1160202

frags 530_R1R2.sorted.bam: coverage 1018

Total Reads: 9989452, Coverage: 1018, minDepth: 102

Confirmed 1160121 of 1160202 bases (99.99%)

Corrected 0 snps; 0 ambiguous bases; corrected 0 small insertions totaling 0 bases, 0 small deletions totaling 0 bases

# Attempting to fix local continuity breaks

LSUCC0530_final_assembly:1-1160202 log:

Finished processing LSUCC0530_final_assembly:1-1160202

Writing updated LSUCC0530_final_assembly_pilon to pilon.fasta

Mean frags coverage: 1018

Mean total coverage: 1018

**Step 7.**

$ checkm lineage_wf -x .fasta -t 16 -f 530_sspacev3_scaffold2 530_sspacev3_in/ 530_sspacev3_out/

CheckM results still predict the scaffold to be 100% complete with 0% contamination, and it clades in the same space with the subclade III taxa. Output:

---------------------------------------------------------------------------------------------------------------------------------------------------------------------------

  Bin Id                       Marker lineage      # genomes   # markers   # marker sets   0    1    2   3   4   5+   Completeness   Contamination   Strain heterogeneity

---------------------------------------------------------------------------------------------------------------------------------------------------------------------------

  530_sspacev3_scaffold2   k__Bacteria (UID2495)      2993        139            83        0   139   0   0   0   0       100.00           0.00               0.00

---------------------------------------------------------------------------------------------------------------------------------------------------------------------------

**Step 8.**

gc_skew

$ gc_skew -f LSUCC0530_final_assembly.fasta

LSUCC0530_final_assembly -> Origin: 640,030 Terminus: 51,160

This final assembly was submitted to IMG (Markowitz *et al.*, 2014) for genome annotation. It is publically available with IMG Taxon ID 2728369501, and at GenBank under accession number CP024034.

**Supplemental Tables and Figures**

**Supplemental Table**

**Table S1** is provided as a spreadsheet, Table_S1.xlsx (hosted with the publication link at http://thethrashlab.com/publications), and contains tabs detailing the AAI vs. synteny analyses; all gene terms for Figure 3; the complete record of osmolyte transport and synthesis genes examined in this study; metadata for samples and genomes used in this study; RPKM calculations; and media recipes. Osmolyte genes were identified using a variety of sources (Agnello *et al.*, 2013, Brill *et al.*, 2011, Bursy *et al.*, 2007, Chen *et al.*, 1998, Curson *et al.*, 2017, Dupont *et al.*, 2004, Empadinhas and da Costa, 2008, García-Estepa *et al.*, 2006, Hagemann and Erdmann, 1994, Hagemann, 2011, Hosie and Poole, 2001, Jorasch *et al.*, 1998, Klähn and Hagemann, 2011, Kronemeyer *et al.*, Lidbury *et al.*, 2014, Lin *et al.*, 2011, Pflüger *et al.*, 2003, Quintero *et al.*, 2001, Reshetnikov *et al.*, 2006, van Alebeek *et al.*, 1992, Walshaw and Poole, 1996, Wood, 2015), indicated in the table.

**Supplemental Figure Legends**

**Figure S1.** Flow cytometry plot of strain LSUCC0530 during the initial experiment by which it was isolated. Side scatter is plotted vs. green fluorescence.

**Figure S2.** Phylogenetic inference of the Alphaproteobacteria, including LSUCC0530 and other reference SAR11 sequences, using 16S rRNA genes. Values at nodes indicate Shimodaira-Hasegawa like values from FastTree2.

**Figure S3.** Box plots of intragenic spacer distributions for subclade IIIa and IIIb (LD12) with Wilcoxon rank-sum results indicated.

**Figure S4.** Metagenomic recruitment to the LSUCC0530 genome scaffold using sequences from Feitsui Reservoir (A) and Lake Gatun (B). Recruitment is plotted according to percent identity of read hit to the genome, which is depicted linearly. HVR2 is visible as the region of very poor recruitment on the left side of each plot, highlighted with the red bars.

**Figure S5.** Phylogenetic tree of *aceA* (isocitrate dehydrogenase) sequences. Scale bar indicates 0.1 changes per position. Values at nodes indicate Shimodaira-Hasegawa like values from FastTree2.

**Figure S6.** Phylogenetic tree of malate synthase sequences. Scale bar indicates 0.1 changes per position. Values at nodes indicate Shimodaira-Hasegawa like values from FastTree2.

**Figure S7.** Gene neighborhood of malate synthase (red, center) in the LSUCC0530 genome and those of a subset of other SAR11 genomes. Conserved region is boxed.

**Figure S8.** Results of growth experiments testing salinity range (A) and temperature range (B). Results are plotted as cell concentration by hour, and are centered at zero as the beginning of logarithmic growth to allow for ease of comparison across multiple independent experiments. Replicates are plotted as separate colors. Replicates showing aberrant growth (e.g., black line in A, 2.9) were not plotted in Figure 4.

**Figure S9.** Relative abundance of the LD12 OTU in coastal samples from the northern Gulf of Mexico. Bar graph indicates OTU7 relative abundance compared with all other SAR11 OTUs, with sites ordered according to increasing salinity. Inset depicts the LD12 OTU7 only data with a linear regression, equation and R^2^ value included, and 95% confidence intervals shaded.

**Figure S10.** Relative abundance of LD12 genome microclusters at 85, 90, 92, 98, and 100% percent identity, for comparison with Figure 5 (95% identity). RPKM values are listed by site, with data aggregated for all genomes in microclusters A-C, according to the key. Colors indicate broad environmental categories.

**Additional Supplemental Information**, including Table S1, alignments, scripts, and density plots for all recruitment analyses, is available with the manuscript link at http://thethrashlab.com/publications.

**References Cited**

Agnello G, Chang LL, Lamb CM, Georgiou G, Stone EM (2013). Discovery of a Substrate Selectivity Motif in Amino Acid Decarboxylases Unveils a Taurine Biosynthesis Pathway in Prokaryotes. *ACS Chemical Biology* **8:** 2264-2271.

Bankevich A, Nurk S, Antipov D, Gurevich AA, Dvorkin M, Kulikov AS *et al.* (2012). SPAdes: A New Genome Assembly Algorithm and Its Applications to Single-Cell Sequencing. *Journal of Computational Biology* **19:** 455-477.

Boetzer M, Henkel CV, Jansen HJ, Butler D, Pirovano W (2011). Scaffolding pre-assembled contigs using SSPACE. *Bioinformatics* **27:** 578-579.

Bolger AM, Lohse M, Usadel B (2014). Trimmomatic: a flexible trimmer for Illumina sequence data. *Bioinformatics* **30:** 2114-2120.

Brill J, Hoffmann T, Bleisteiner M, Bremer E (2011). Osmotically Controlled Synthesis of the Compatible Solute Proline Is Critical for Cellular Defense of Bacillus subtilis against High Osmolarity. *J Bacteriol* **193:** 5335-5346.

Brown CT, Hug LA, Thomas BC, Sharon I, Castelle CJ, Singh A *et al.* (2015). Unusual biology across a group comprising more than 15% of domain Bacteria. *Nature* **523:** 208-211.

Bursy J, Pierik AJ, Pica N, Bremer E (2007). Osmotically Induced Synthesis of the Compatible Solute Hydroxyectoine Is Mediated by an Evolutionarily Conserved Ectoine Hydroxylase. *Journal of Biological Chemistry* **282:** 31147-31155.

Chen L, Spiliotis ET, Roberts MF (1998). Biosynthesis of Di-myo-inositol-1,1'-phosphate, a novel osmolyte in hyperthermophilic archaea. *J Bacteriol* **180:** 3785-3792.

Curson ARJ, Liu J, Martínez A, Green RT, Chan Y, Carrión O *et al.* (2017). Dimethylsulfoniopropionate biosynthesis in marine bacteria and identification of the key gene in this process. *Nature Microbiology* **2:** 17009.

Dupont L, Garcia I, Poggi M-CC, Alloing G, Mandon K, Le Rudulier D (2004). The Sinorhizobium meliloti ABC transporter Cho is highly specific for choline and expressed in bacteroids from Medicago sativa nodules. *J Bacteriol* **186:** 5988-5996.

Empadinhas N, da Costa M (2008). Osmoadaptation mechanisms in prokaryotes: distribution of compatible solutes. *International Microbiology*.

García-Estepa R, Argandoña M, Reina-Bueno M, Capote N, Iglesias-Guerra F, Nieto JJ *et al.* (2006). The ectD Gene, Which Is Involved in the Synthesis of the Compatible Solute Hydroxyectoine, Is Essential for Thermoprotection of the Halophilic Bacterium Chromohalobacter salexigens. *J Bacteriol* **188:** 3774-3784.

Hagemann M, Erdmann N (1994). Activation and pathway of glucosylglycerol synthesis in the cyanobacterium Synechocystis sp. PCC 6803. *Microbiology* **140:** 1427-1431.

Hagemann M (2011). Molecular biology of cyanobacterial salt acclimation. *FEMS Microbiology Reviews* **35:** 87-123.

Hosie A, Poole P (2001). Bacterial ABC transporters of amino acids. *Research in Microbiology* **152:** 259-270.

Hunt M, Kikuchi T, Sanders M, Newbold C, Berriman M, Otto TD (2013). REAPR: a universal tool for genome assembly evaluation. *Genome Biol* **14:** 1-10.

Jorasch P, Wolter FP, Zähringer U, Heinz E (1998). A UDP glucosyltransferase from Bacillus subtilis successively transfers up to four glucose residues to 1,2‐diacylglycerol: expression of ypfP in Escherichia coli and structural analysis of its reaction products. *Molecular Microbiology* **29:** 419-430.

Klähn S, Hagemann M (2011). Compatible solute biosynthesis in cyanobacteria. *Environmental Microbiology* **13:** 551-562.

Kronemeyer W, Peekhaus N, Krämer R, Sahm H, Eggeling L (1995). Structure of the gluABCD cluster encoding the glutamate uptake system of Corynebacterium glutamicum. *J Bacteriol* **177:** 1152-1158.

Li H, Durbin R (2009). Fast and accurate short read alignment with Burrows–Wheeler transform. *Bioinformatics* **25:** 1754-1760.

Lidbury I, Murrell JC, Chen Y (2014). Trimethylamine N-oxide metabolism by abundant marine heterotrophic bacteria. *Proc Natl Acad Sci USA* **111:** 2710-2715.

Lin H, Jiang P, Zhang J, Wang J, Qin S, Sun S (2011). Genetic and Marine Cyclonic Eddy Analyses on the Largest Macroalgal Bloom in the World. *Environ Sci Technol* **45:** 5996-6002.

Markowitz VM, Chen IMA, Palaniappan K, Chu K, Szeto E, Pillay M *et al.* (2014). IMG 4 version of the integrated microbial genomes comparative analysis system. *Nucleic Acids Research* **42:** D560-567.

Parks DH, Imelfort M, Skennerton CT, Hugenholtz P, Tyson GW (2015). CheckM: assessing the quality of microbial genomes recovered from isolates, single cells, and metagenomes. *Genome Research* **25:** 1043-1055.

Pflüger K, Baumann S, Gottschalk G, Lin W, Santos H, Müller V (2003). Lysine-2,3-aminomutase and ß-lysine acetyltransferase genes of methanogenic archaea are salt induced and are essential for the biosynthesis of Ne-acetyl-ß-lysine and growth at high salinity. *Appl Environ Microbiol* **69**.

Quintero Ma, Montesinos Ma, Herrero A, Flores E (2001). Identification of Genes Encoding Amino Acid Permeases by Inactivation of Selected ORFs from the Synechocystis Genomic Sequence. *Genome Research* **11:** 2034-2040.

Reshetnikov AS, Khmelenina VN, Trotsenko YA (2006). Characterization of the ectoine biosynthesis genes of haloalkalotolerant obligate methanotroph “Methylomicrobium alcaliphilum 20Z”. *Archives of Microbiology* **184:** 286-297.

van Alebeek G-J, Hermans JMH, Keltjens JT, Vogels GD (1992). Quantification of intermediates involved in the cyclic 2,3-diphosphoglycerate metabolism of methanogenic bacteria by ion-exchange chromatography. *Journal of Chromatography A* **606:** 65-71.

Walker BJ, Abeel T, Shea T, Priest M, Abouelliel A, Sakthikumar S *et al.* (2014). Pilon: An Integrated Tool for Comprehensive Microbial Variant Detection and Genome Assembly Improvement. *PLOS ONE* **9**.

Walshaw DL, Poole PS (1996). The general l‐amino acid permease of Rhizobium leguminosarum is an ABC uptake system that also influences efflux of solutes. *Molecular Microbiology* **21:** 1239-1252.

Wood JM (2015). Bacterial responses to osmotic challenges. *The Journal of General Physiology* **145:** 381-388.
