# Supplementary figures and images for: Cultivation and genomics of the first freshwater SAR11 (LD12) isolate
Source: ISME J. 2018 Mar 29;12(7):1846–60. doi: 10.1038/s41396-018-0092-2 (PMC6018831; doi:10.1038/s41396-018-0092-2)

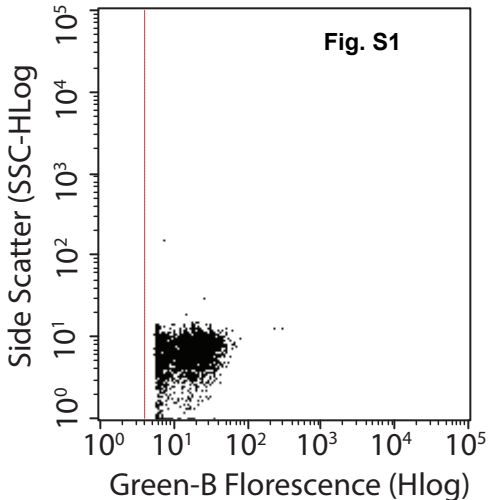

Supplement: Supplementary file 2 — Figure S1(PDF 383 kb) [file 41396_2018_92_MOESM2_ESM.pdf]

Fig. S2

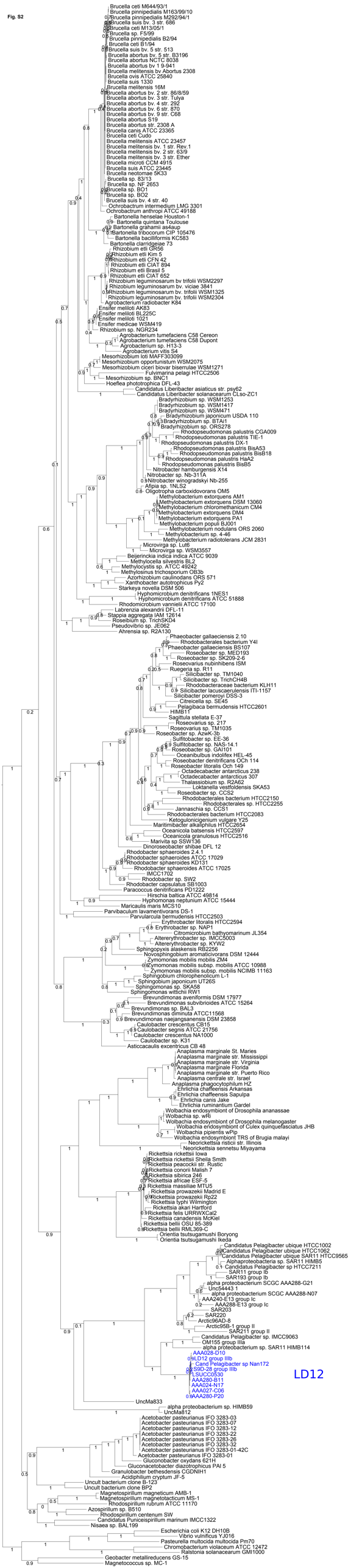

LD12

Supplement: Supplementary file 3 — Figure S2(PDF 125 kb) [file 41396_2018_92_MOESM3_ESM.pdf]

**Fig. S3 SAR11 intergenic spacer distribution**

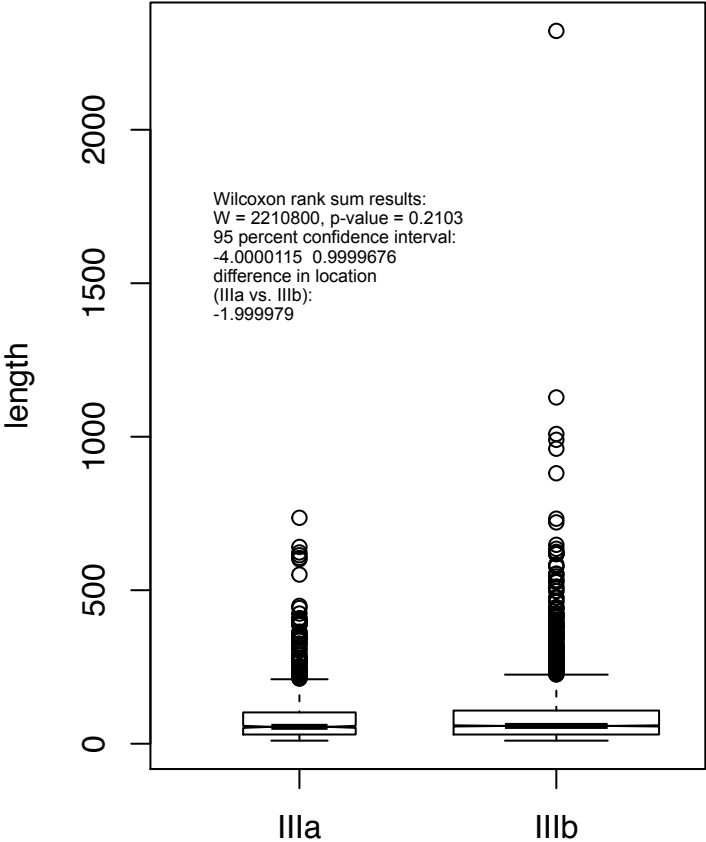

Supplement: Supplementary file 4 — Figure S3(PDF 135 kb) [file 41396_2018_92_MOESM4_ESM.pdf]

**a****Feitsui Reservoir-July 2007**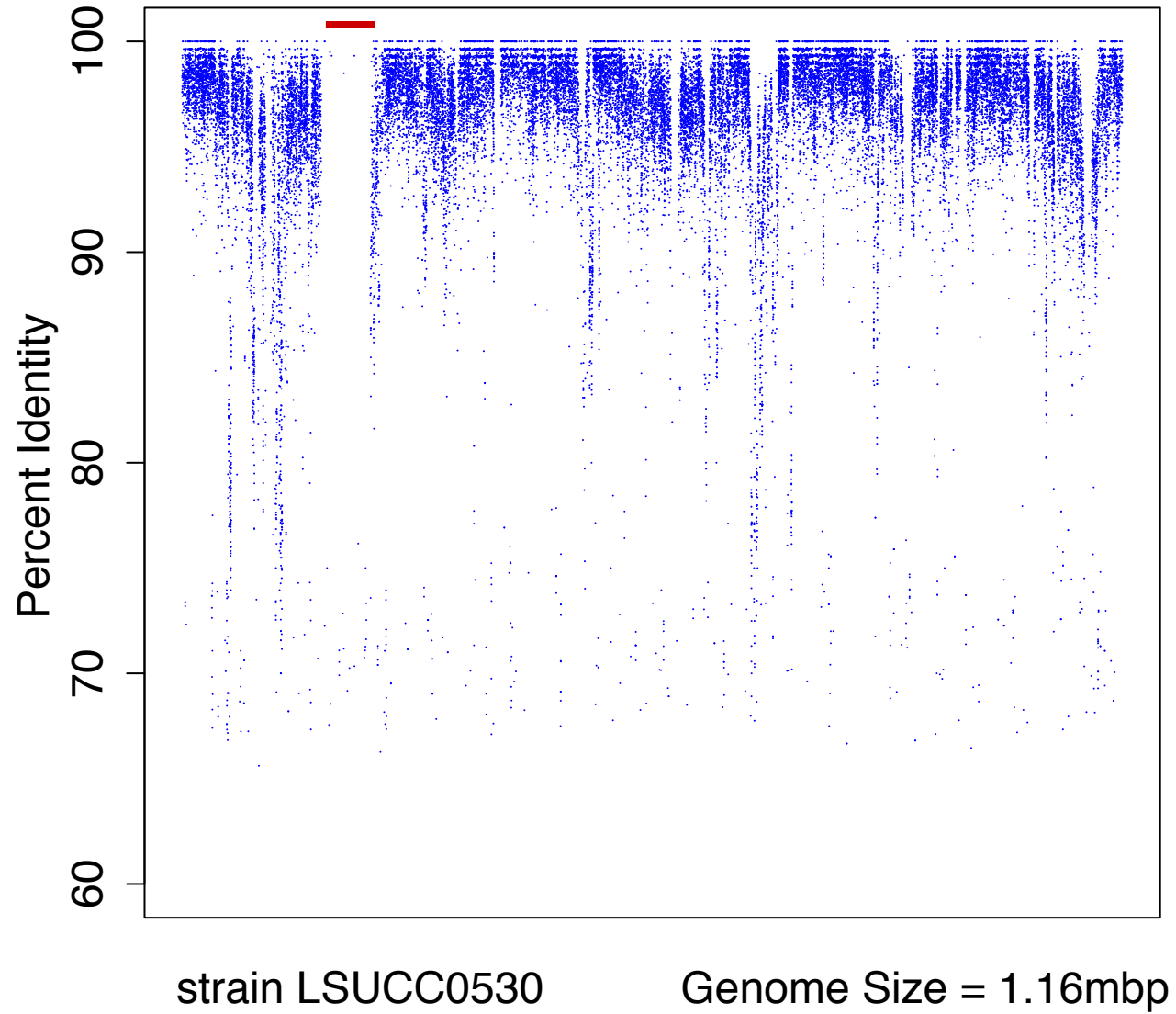**b****Lake Gatun**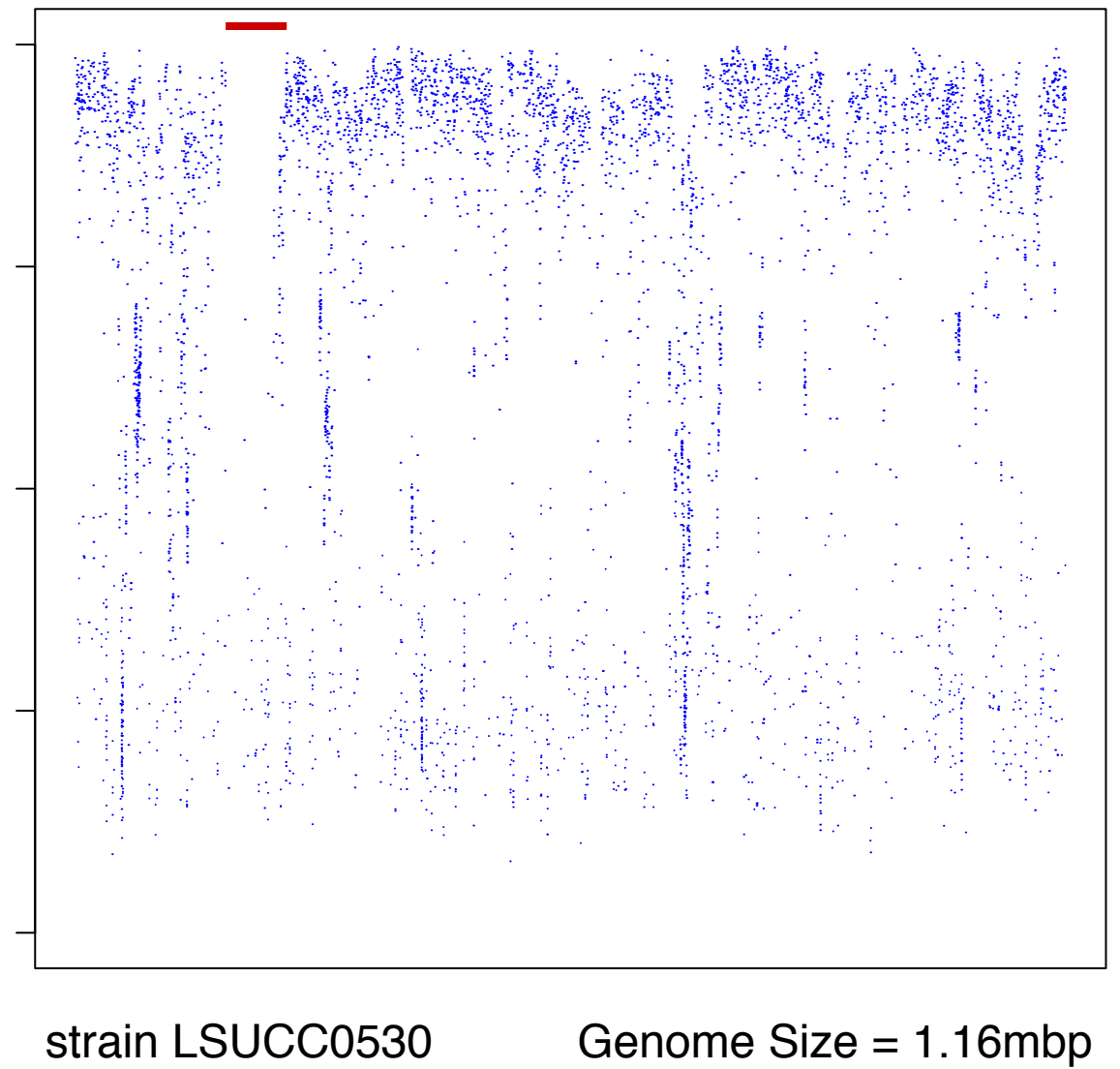

Supplement: Supplementary file 5 — Figure S4(PDF 1080 kb) [file 41396_2018_92_MOESM5_ESM.pdf]

Fig. S5

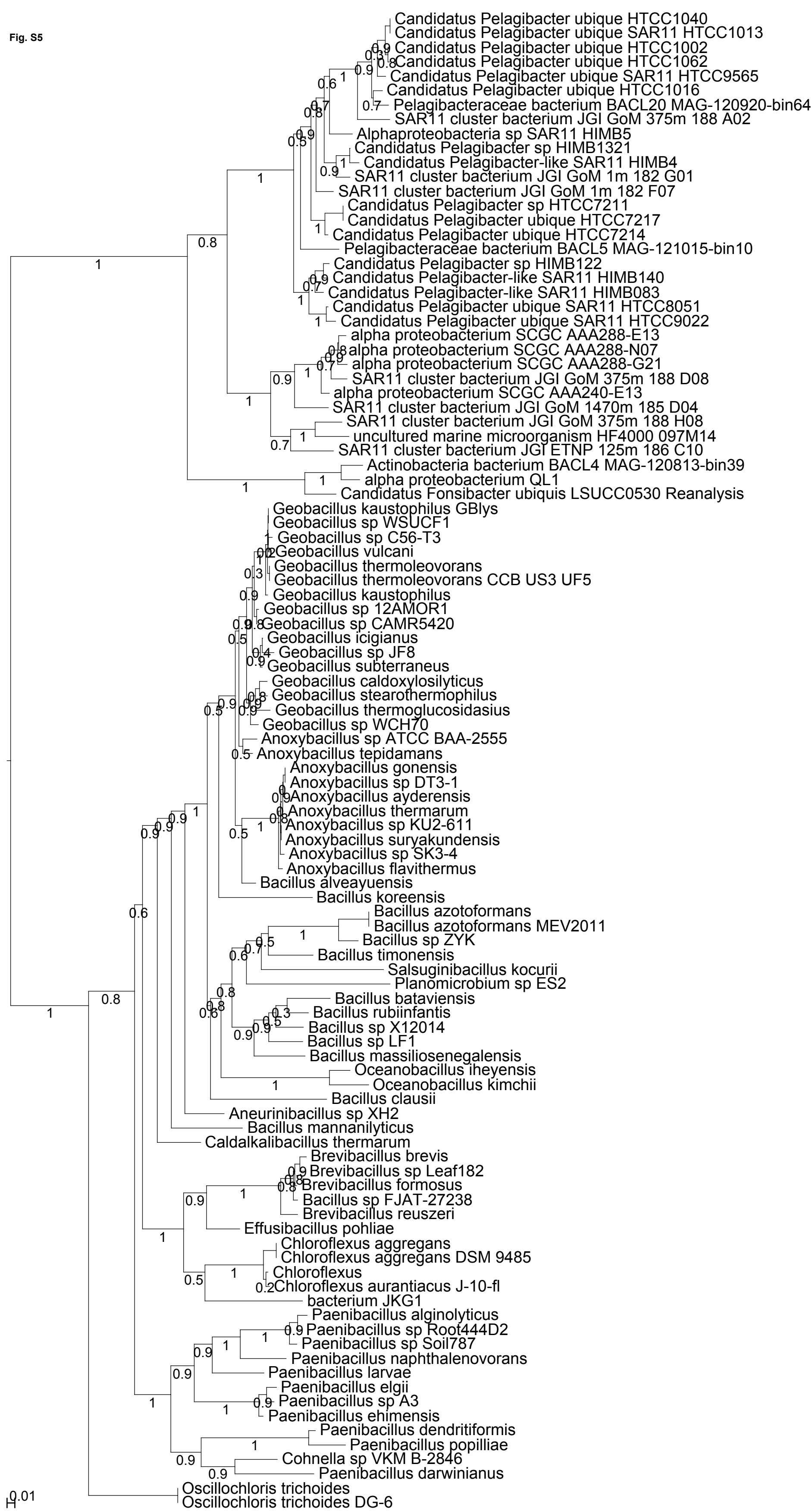

Supplement: Supplementary file 6 — Figure S5(PDF 117 kb) [file 41396_2018_92_MOESM6_ESM.pdf]

Fig. S6

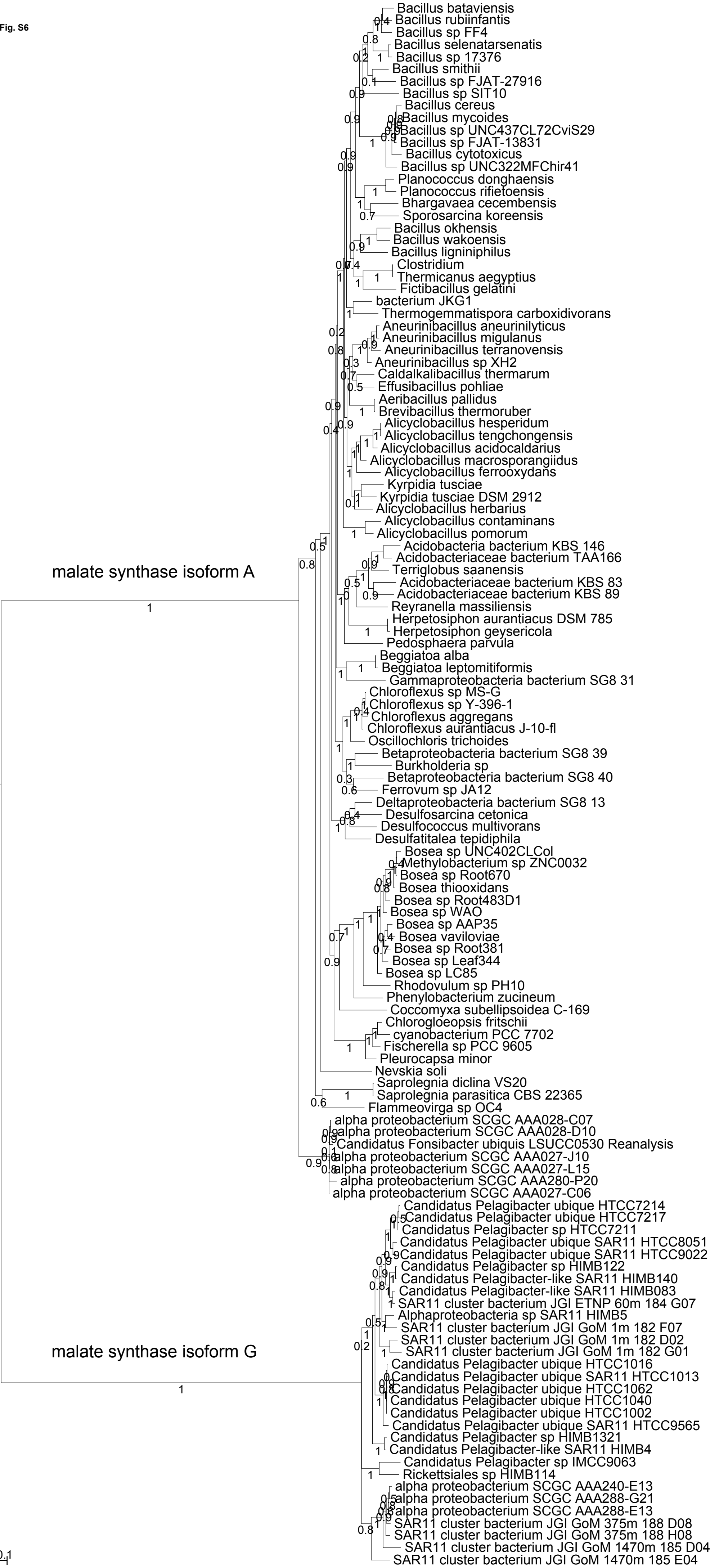

Supplement: Supplementary file 7 — Figure S6(PDF 147 kb) [file 41396_2018_92_MOESM7_ESM.pdf]

Fig. S7

malate synthase (red)

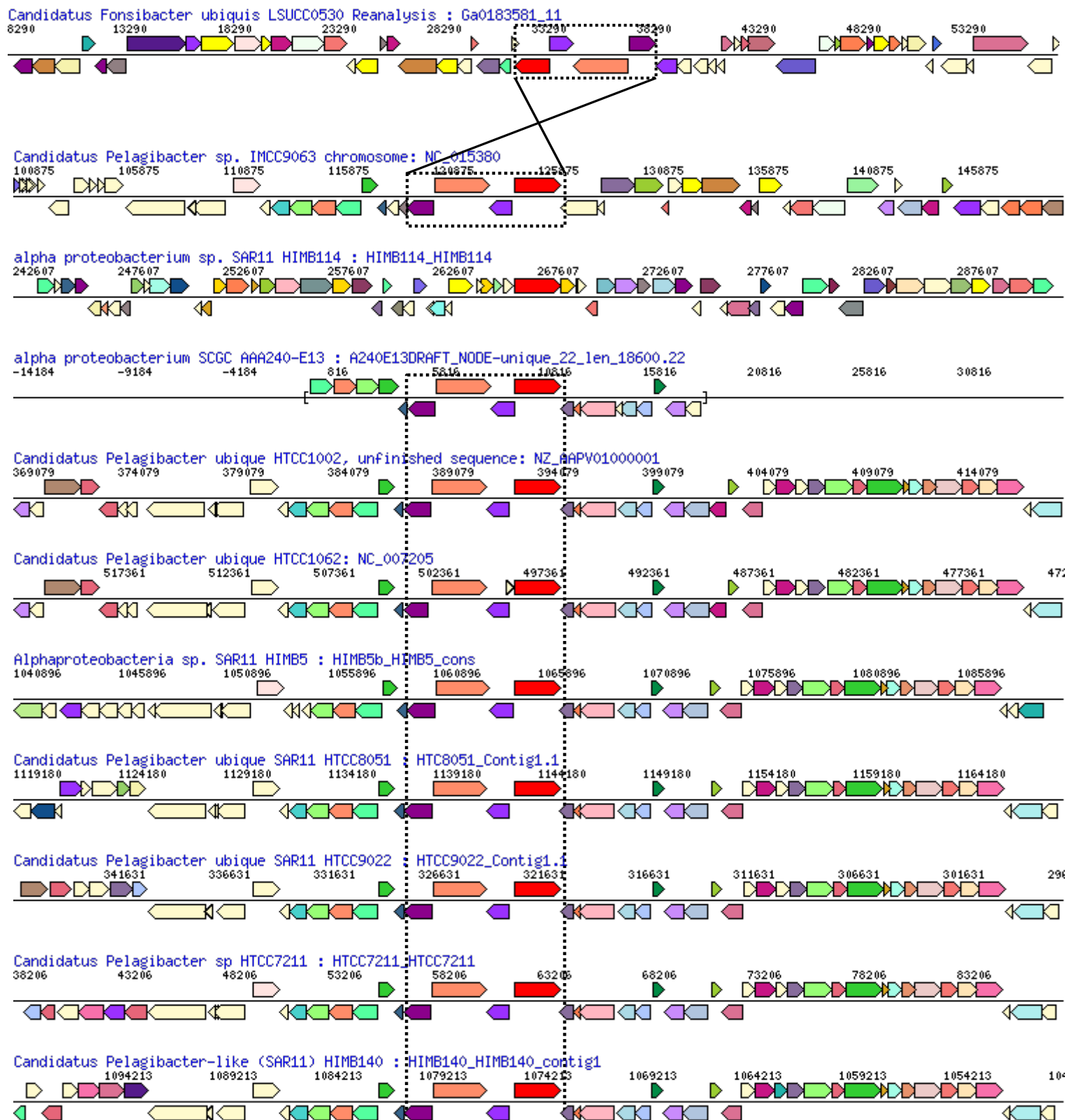

Supplement: Supplementary file 8 — Figure S7(PDF 126 kb) [file 41396_2018_92_MOESM8_ESM.pdf]

Fig. S8

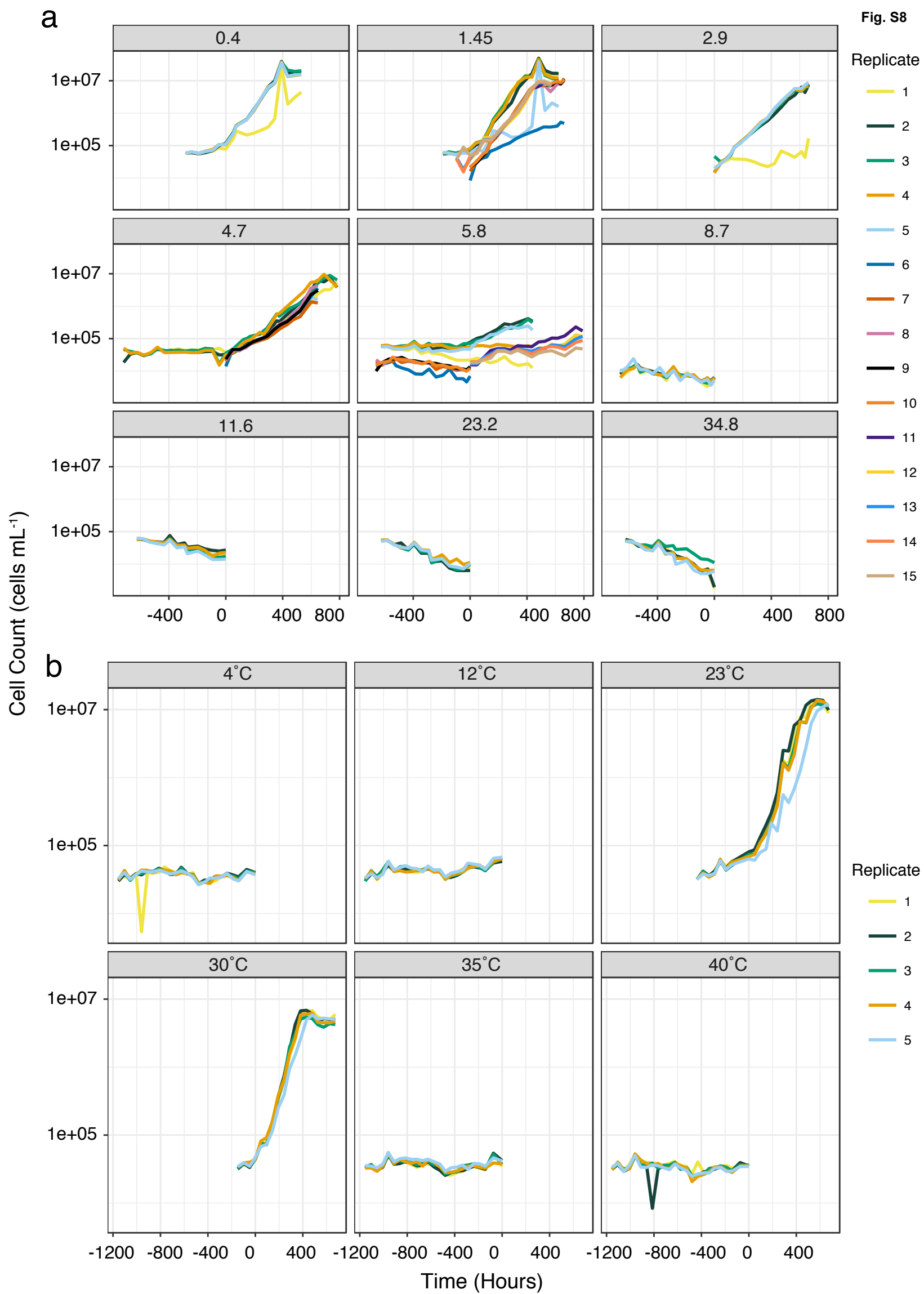

Supplement: Supplementary file 9 — Figure S8(PDF 271 kb) [file 41396_2018_92_MOESM9_ESM.pdf]

Fig. S9

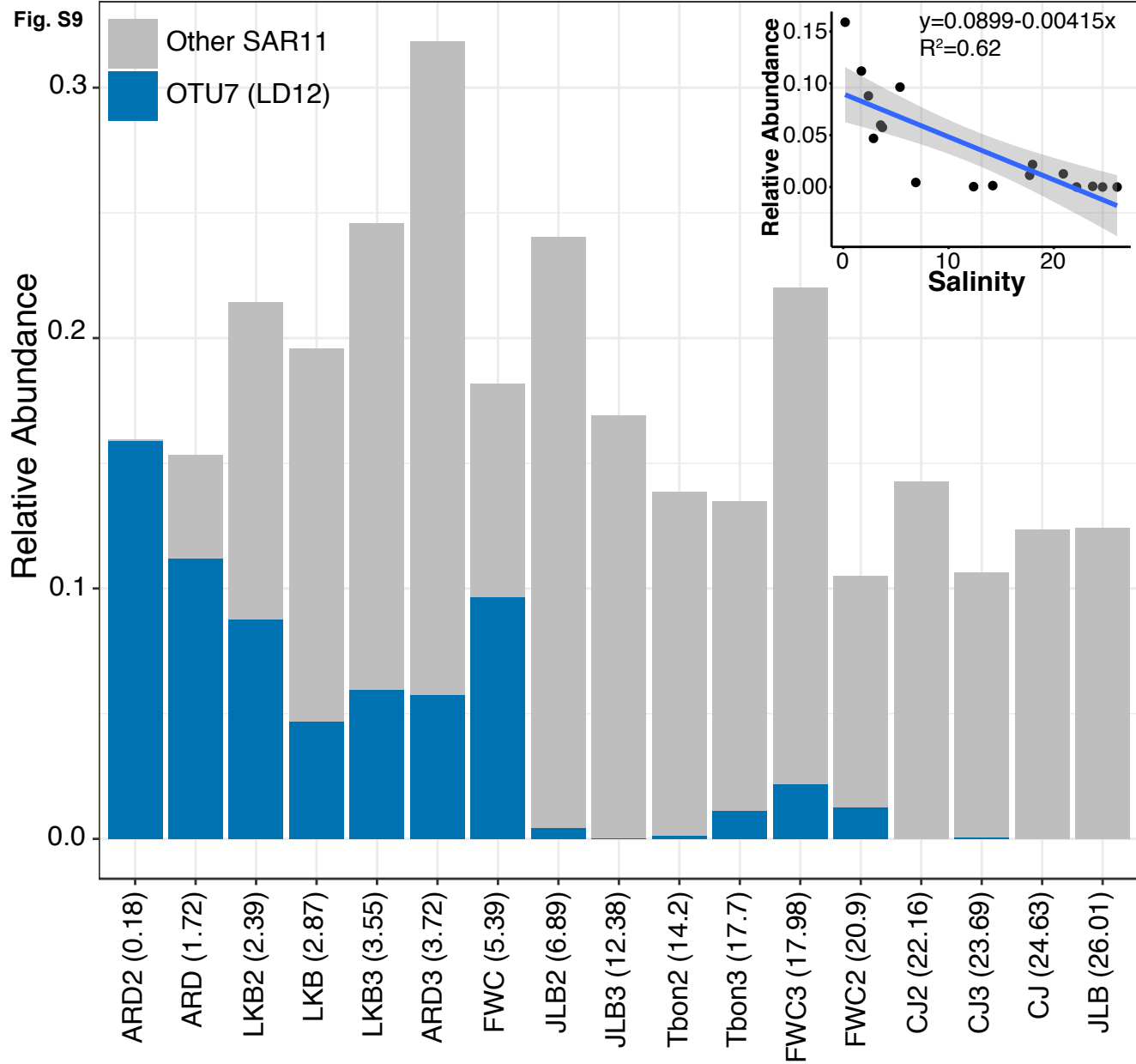

Supplement: Supplementary file 10 — Figure S9(PDF 250 kb) [file 41396_2018_92_MOESM10_ESM.pdf]

Fig. S10

Environment

- Baltic Sea
- Coastal
- Rivers and Lakes

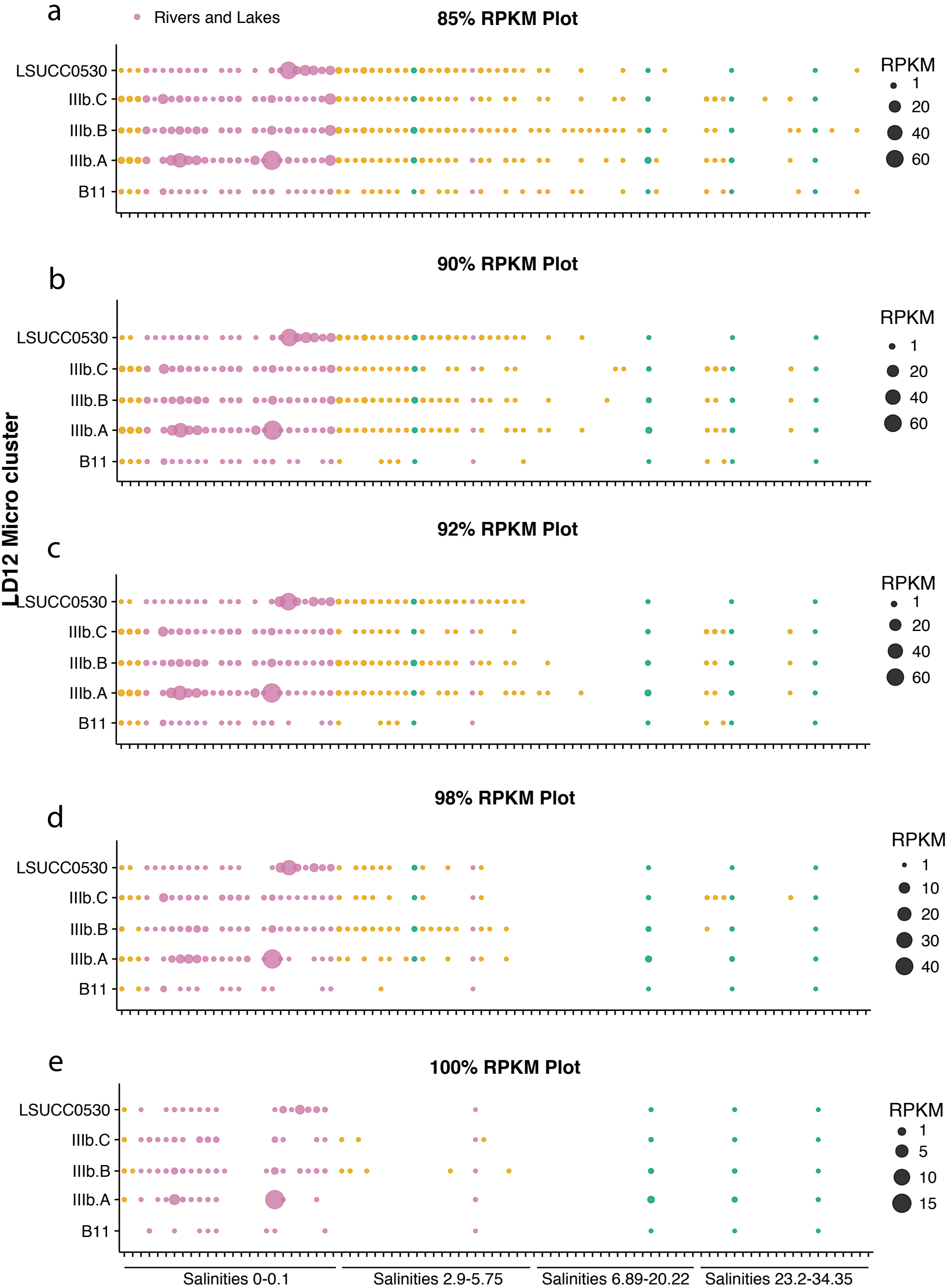

Supplement: Supplementary file 11 — Figure S10(PDF 1437 kb) [file 41396_2018_92_MOESM11_ESM.pdf]
